# Editorial: Autoimmunity and Chronic Inflammation in Early Life

**DOI:** 10.3389/fimmu.2021.761160

**Published:** 2021-09-09

**Authors:** Ravi Misra, Jennifer Konopa Mulligan, Sarah Rowland-Jones, Michael Zemlin

**Affiliations:** ^1^Department of Pediatrics, The University of Rochester Medical Center, Rochester, NY, United States; ^2^Division of Pulmonary, Critical Care & Sleep Medicine, University of Florida, Gainesville, FL, United States; ^3^Viral Immunology Unit, Nuffield Department of Medicine, Oxford, United Kingdom; ^4^Department for General Pediatrics and Neonatology, Saarland University, Homburg, Germany

**Keywords:** autoimmunity, pediatrics - children, immune development and maturation, early life origin of disease, chronic inflammation

## Introduction

Non-communicable diseases such as cardiovascular diseases, metabolic disease and chronic inflammatory diseases are often attributed to an interplay between genetic predispositions and imprinting mechanisms early in life. In a simplified concept, the fetal development is characterized by the acquisition of immuno-tolerance towards maternal and self-antigens, whereas the neonatal period reflects the acquisition of immune-defense against potentially harmful environmental antigen. Immune development involves a complex cross talk of immune cells in various organs that is influenced by environmental antigen (1–3). During infancy and childhood, autoimmune diseases and chronic inflammation coincide with an exponential diversification of the adaptive immune system, causing potentially life-long consequences. Interestingly, the earlier hypothesis of an “immunodeficiency of immaturity” had to be partially revised since it has become clear that inflammatory states in the neonate, e.g. in the context of sepsis, represent a lack of controlling inflammation rather than a failure to mount inflammation. Thus, hyperinflammatory states can occur even in the very immature organism and can lay the ground for autoimmunity or various conditions of chronic inflammation. This knowledge may affect therapeutic approaches.

In this Research Topic, we have called for publications that relate to clinical or molecular aspects of aberrant immune responses in pediatric patients. Here we briefly present the 12 contributions that comprise six Original Research articles, three (mini) reviews and three case reports. The contributions can be grouped into three sections:

1. Clinical manifestations of early autoimmune and chronic inflammatory diseases
2. From molecular mechanisms to autoimmune phenotypes
3. The role of B- and T cells for autoimmune diseases and chronic inflammation in early life

## Clinical Manifestations of Early Autoimmune and Chronic Inflammatory Diseases

In a single center overview, Wang et al. present the genetic and clinical characteristics of 79 pediatric patients with monogenetic autoimmune diseases. The patients were affected with 18 different diagnoses that can be grouped into inflammasomopathies (42%), non-inflammasome related conditions (48%) and type 1 interferonopathies (10%). 76% of the patients presented with skin disorders, making this the most common clinical clue to autoimmune diseases. In contrast, Chinello et al. address a new complication in a rare autoimmune disease by presenting a pediatric patient with lipopolysaccharide-responsive-beige-linked-anchor-protein (LRBA) deficiency that developed acute cervical longitudinally transverse myelitis. Chen et al. report a patient with symptoms of intestinal Behcet’s disease that was associated with novel heterozygous mutation in the TNFAIP gene, potentially linking intestinal Behcet’s disease with Haploinsufficiency A20.

## From Molecular Mechanisms to Autoimmune Phenotypes

Haploinsufficiency A20 (HA20) has recently been identified as one potential molecular mechanism of autoimmunity. In an analysis of 89 patients with this rare condition, Chen et al. present the typical clinical manifestations associated with HA20: Recurrent oral ulcers and fever episodes, gastrointestinal and genital ulcers as well as skin lesions are the hallmarks that can help clinicians to initiate targeted diagnostics in patients that often suffer a long odyssey prior to a correct diagnosis and therapy. Another diagnostic “chameleon”, systemic lupus erythematosus has been addressed by Motwani et al.: In an elegant series of experiments, the authors could rule out a contribution of the cGAS-STING pathway to the clinical symptoms in a murine model of chronic SLE, thus questioning the potential of novel cGAS-STING directed therapeutic approaches. As shown by Wang et al. in this Research Topic, inflammasomopathies represent a major percentage of autoimmune diseases, including type 1 diabetes. In extension of these observations, Sun et al. review the literature on the assembly and function of the NLRP3 inflammasome and its role in type 1 diabetes.

## The Role of B- and T Cells for Autoimmune Diseases and Chronic Inflammation in Early Life

While Sun et al. address the role of the NLRP3 inflammasome in diabetes, Shapiro et al. address T cell mediated mechanisms in the same disease: The severity of type 1 diabetes was reduced in CD226 deficient non-obese diabetic (NOD) mice. Using adoptive transfer experiments and other techniques, the authors could attribute this phenomenon to the impaired activation of peripheral T cells, which is mediated by CD226. Foth et al. contribute a review on the T cell repertoire during ontogeny and characteristic aberrations in inflammatory disorders. Ultimately, this relates to the unanswered question of whether the T cell is rather a bystander or a culprit during the development of autoimmunity and chronic inflammatory diseases. The small but still enigmatic subset of IL-17 expressing TH cells plays a key role in the pathogenesis of chronic mucocutaneous candidiasis in children and are diminished in other autoimmune diseases such as hyper IgE syndrome. Shamriz et al. give an overview on the immune mechanisms, current management and novel targeted therapies for monogenic mucocutaneous candidiasis with a focus on TH 17 cells. Two articles of this Research Topic focus on the immunologic and inflammatory aspects of lung disease: Bhattacharya et al. present transcriptomic profiles of purified CD8+ T cells from preterm neonates at the time of discharge. By identifying signaling pathways that differ between infants with and without bronchopulmonary dysplasia this study sheds new light into the patho-mechanisms of this condition, which remains a major cause of long-term morbidity in preterm neonates. Hwang et al. report that inducible Bronchus-Associated Lymphoid Tissue (iBALT) attenuates the allergic airway inflammation in a mouse model by sequestration of effector T cells. On the other hand, Breville et al. present a case report suggesting a role of IgG4 in severe complement-mediated thrombotic microangiopathy in a patient with IgG4 related disease. Interestingly, a genetic predisposition might enhance the susceptibility to form inhibitory anti-Factor H IgG4 antibodies.

## Concluding Remarks

The article collection of the Research Topic gives an up-to-date overview on the clinical manifestations and molecular mechanisms of autoimmunity and chronic inflammation in early life. Thus, in the jungle of “diagnostic chameleons”, clinicians may find important hints for linking characteristic symptoms with autoimmune diseases. On the other hand, basic researchers find a thorough overview of the current knowledge on disease mechanism that should inspire research projects in the field of diagnostics and novel therapeutic concepts.

## Author Contributions

MZ wrote the first draft of the manuscript. All authors contributed to the article and approved the submitted version.

## Funding

This study was supported by BMBF PRIMAL study (01GL1746D) to MZ.

## Conflict of Interest

The authors declare that the research was conducted in the absence of any commercial or financial relationships that could be construed as a potential conflict of interest.

## Publisher’s Note

All claims expressed in this article are solely those of the authors and do not necessarily represent those of their affiliated organizations, or those of the publisher, the editors and the reviewers. Any product that may be evaluated in this article, or claim that may be made by its manufacturer, is not guaranteed or endorsed by the publisher.
